# Efficient Conversion of Lignin to Aromatics via Catalytic Fast Pyrolysis over Niobium-Doped HZSM-5

**DOI:** 10.3390/molecules28104245

**Published:** 2023-05-22

**Authors:** Zhen Li, Huihui Zhang, Deshi Yang, Zhipeng Hu, Fengqiang Wang, Zhijun Zhang

**Affiliations:** Key Laboratory of Bio-Based Material Science and Technology (Ministry of Education), Northeast Forestry University, Harbin 150040, Chinazhhhhhh@nefu.edu.cn (H.Z.); dsyang@nefu.edu.cn (D.Y.); zpenghu@nefu.edu.cn (Z.H.); fqw@nefu.edu.cn (F.W.)

**Keywords:** catalytic fast pyrolysis, alkali lignin, Nb-doped HZSM-5, deoxygenation

## Abstract

A niobium-doped HZSM-5 (H[Nb]ZSM-5) was prepared by a hydrothermal synthesis method. The morphology, phase structure, composition, pore structure, and acid content of the catalyst were characterized using a series of analysis techniques such as scanning electron microscope (SEM), energy-dispersive X-ray (EDX), X-ray diffraction (XRD), X-ray photoelectron spectroscopy (XPS), nitrogen adsorption-desorption, and temperature programmed desorption measurements (NH_3_-TPD). The H[Nb]ZSM-5 catalyst fully remained within the crystal framework and pore structure of HZSM-5. Meanwhile, introduction of niobium (V) endowed the catalyst with both Lewis acid and Bronsted acid sites. Catalytic fast pyrolysis (CFP) of alkali lignin was carried out through a pyrolysis and gas chromatography-mass spectrometry (Py-GC/MS) at 650 °C and atmospheric pressure. The results indicated that H[Nb]ZSM-5 can efficiently and selectively convert lignin into monoaromatic hydrocarbons (MAHs), compared to the control HZSM-5. Catalyzed by H[Nb]ZSM-5, the content of MAHs and aliphatic hydrocarbons reached 43.4% and 20.8%, respectively; while under the catalysis of HZSM-5, these values were 35.5% and 3.2%, respectively. H[Nb]ZSM-5 remarkably lowered the phenol content to approximately 2.8%, which is far lower than the content (24.9%) obtained under HZSM-5 catalysis.

## 1. Introduction

Biomass is an environmentally friendly and renewable energy source that has the potential to replace traditional petroleum resources. Lignin is the most abundant renewable aromatic polymer in nature [1], with up to 50 billion tons formed annually through photosynthesis on earth [2]. Approximately 50 million tons of them were produced as the by-product of traditional pulp and paper industry and biorefineries per year [3]. However, the complex chemical structure of lignin makes it difficult to be utilized directly. Typically, more than 98% of these lignin waste is directly burned for energy [4], resulting in a low utilization efficiency. Thus, it is necessary to utilize lignin waste to improve the comprehensive utilization efficiency of biomass resources.

The lignin depolymerization process [5] can convert lignin into high-quality biofuels and chemicals. It is beneficial to improve the economic efficiency of pulp and bio-refining industries [6]. Currently, the depolymerization methods of lignin mainly include thermochemical conversion, mechanical depolymerization, catalytic fast pyrolysis (CFP), and biodegradation [7]. Among them, CFP refers to a process that produces bio-oil, non-condensable gas, and bio-char by heating biomass pellets in the presence of a catalyst in an oxygen-free or anoxic environment [8]. It is a relatively efficient biomass conversion technology that has been widely used for converting lignin into aromatic compounds [9]. Development of an efficient catalyst with good performance is the core of this technology. Commonly, catalysts used in CFP technology include metal salts [10,11], metal oxides [12], zeolite molecular sieves [13,14], etc. Zeolite molecular sieves have become one of the most promising catalysts in CFP technology due to their excellent hydrothermal stability, controllable acidic strength, shape selectivity, and other properties [15]. Among them, HZSM-5 has attracted wide attention due to its unique pore structure, excellent shape-selective catalytic ability, and good deoxygenation performance. It has been used for catalytic thermal cracking of lignin into bio-oil rich in monocyclic aromatic hydrocarbon products [16]. However, due to the hydrogen-deficient and oxygen-rich nature of lignin and its fragments, coke is always generated during pyrolysis. This can cover acidic sites and block pores, leading to a decrease in catalyst activity and selectivity. In addition, lignin pyrolysis products contain a large number of phenolics, which can rapidly deactivate the molecular sieve [17]. Improper acidity of the molecular sieve is another key reason for both coke formation and low catalytic efficiency [18]. Although the catalyst activity can be restored through oxidative regeneration, low oil yield from lignin pyrolysis caused by coking is a major obstacle to industrialization of this process [19,20]. Therefore, modification of the zeolite catalyst is necessary to obtain a suitable pore structure and an appropriate acid strength and molecular content.

Presently, HZSM-5 modification methods include alkali treatment, impregnation, ion exchange, hydrothermal synthesis, etc. Among these methods, introduction of metals such as Zn [21], Fe [22], Ga [23], Mo [24], and La [25] into the molecular sieve to prepare the bimetallic catalyst (Me/HZSM-5) has been reported in catalytic pyrolysis of lignin and lignin derivatives. This proves that the modified catalyst retains the shape-selective catalytic ability of HZSM-5; meanwhile, addition of a metal active site promotes phenol deoxidation, thus, improved the yield of monoaromatics. Moreover, the synergistic catalytic action of metal sites and Brønsted acid sites reduces the coking and deactivation of the catalyst to some extent [26]. Most of these modified HZSM-5 catalysts were prepared by a impregnation method; the metal active sites were supported on the outer surface of zeolite or in the micropore channels [27]. This increased the diffusion resistance of reactants and products, and prevented the contact between the raw materials and the active site on the catalyst. The metal loaded on the surface of molecular sieves is prone to detachment, leading to a decrease in the catalyst performance. Compared with the impregnation method, hydrothermal synthesis can give a better dispersion of metals and improve the stability of the catalyst [28]. Metal species (Sn, Fe, and Zn) have been incorporated with HZSM-5 to generate H[Me, Al]ZSM-5 using this method and it has been used in methanol aromatization reactions. The results have shown that the highly dispersed metals were beneficial to improve both the yield of mono-aromatic hydrocarbons and the catalyst stability [29,30].

In recent years, owing to the special electronic structure Nb_5c_, metal Nb-modified molecular sieves have attracted great attention for the effective catalytic cracking of C-O bonds [31,32]. Yang [31] et al. synthesized NbAlS-1 by integrating Nb (V) and Al (III) active sites into an MFI-type zeolite framework. NbAlS-1 showed strong water resistance and could quantitatively convert γ-valerolactone aqueous solution to butene at atmospheric pressure. In addition, a synergistic catalytic effect of Nb (v) sites and Brønsted acid sites in promoting C-O bond breaking was found. Wang [32] et al. prepared a Nb-doped SBA-15 molecular sieve by hydrothermal synthesis and applied it in 2, 5-dimethyltetrahydrofuran hydrodeoxygenation to prepare hexane. This proved that the Nb active sites with a low coordination number could effectively activate a C-O bond in the hydrodeoxidation reaction. However, preparing a H[Nb]ZSM-5 bifunctional catalyst by hydrothermal synthesis and using it in catalytic pyrolysis of lignin has rarely been reported.

In this study, a new type of bifunctional niobium-doped ZSM-5 (H[Nb]ZSM-5) was prepared using the hydrothermal synthesis method. Then, the as-prepared catalyst was characterized by X-ray powder diffraction (XRD), physical adsorption, NH_3_-TPD, and X-ray photoelectron spectroscopy (XPS). Finally, the lignin was pyrolyzed using pyrolysis gas chromatography-mass spectrometry (Py-GC/MS), and the effect of H[Nb]ZSM-5 catalyst on the production of light aromatic hydrocarbons (benzene-toluene-xylene mixture, BTX) was studied using nest HZSM-5 as the control. The catalyst stability as well as carbon/coke accumulation behavior were evaluated.

## 2. Results and Discussion

### 2.1. Catalyst Synthesis

The hydrothermal synthesis process of Nb doped H-ZSM-5 (H[Nb]ZSM-5) was systematically studied by changing the crystallization temperature, crystallization time, alkalinity of feed liquid, and niobium source (Supplementary Figures S1–S4). With the aim of producing catalysts with high relative crystallinity, the optimal synthesis conditions were obtained as follows: a silica-alumina ratio of 11, an initial gel pH of 12, a crystallization temperature of 170 °C, and a crystallization time of 72 h. The catalysts involved in the following were all obtained under the optimal conditions mentioned above unless otherwise specified.

### 2.2. Catalyst Characterizations

#### 2.2.1. XRD Analysis

**Figure 1:**
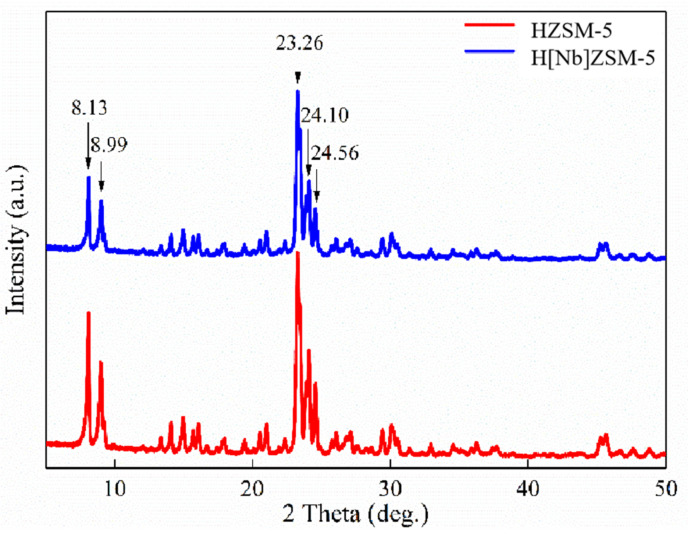
XRD patterns of the catalysts.

Pure phases of both HZSM-5 and H[Nb]ZSM-5 with relatively high crystallinity were synthesized at the optimal conditions mentioned above and determined by XRD (Figure 1). Both HZSM-5 and H[Nb]ZSM-5 showed the main diffraction peaks of HZSM-5 at 2θ = 8.13°, 8.99°, 23.26°, 24.10°, and 24.56°, which are typical of an MFI topological structure crystal plane [33]. This indicated that the crystal structure of HZSM-5 remained intact after the addition of Nb metal. However, no crystalline phase containing niobium oxide was observed in the H[Nb]ZSM-5 catalyst. This might be due to the low loading and high dispersing of Nb atoms, for example, in the form of an nanoscale amorphous phase or highly nanocrystalline phase [24]. Compared with the reference HZSM-5 prepared at same conditions, the relative crystallinity of H[Nb]ZSM-5 is lower (approximately 87.1%), which may be related to the dispersion of Nb source on HZSM-5 (Table 1).

#### 2.2.2. Physical Adsorption Analysis

**Figure 2:**
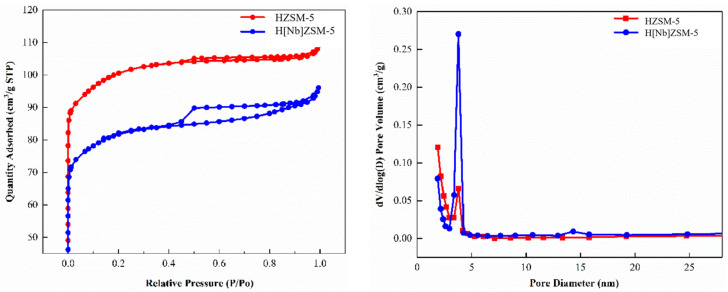
N_2_ adsorption-desorption isotherms and pore size distribution curves of catalysts.

Figure 2 shows the N_2_ adsorption-desorption isotherms and pore size distribution curves of the HZSM-5 and H[Nb] ZSM-5 catalysts. According to the classification of IUPAC, the isotherms of these two catalysts are IV type [34], indicating that the addition of Nb had little effect on the texture of molecular sieves. At very low relative pressures (P/P_0_ = 0–0.01), the isotherms of both HZSM-5 and H[Nb]ZSM-5 catalysts exhibited a sharp increase, indicating the existence of a microporous structure in them [25]. When the relative pressure increases to 0.4–0.9, a noticeable H_4_-type hysteresis ring appeared, which was caused by capillary condensation (multilayer adsorption of single molecular layers) [26]. The pore size distributions of HZSM-5 and H[Nb]ZSM-5 catalysts were between 2–5 nm.

The specific surface area, average pore diameter, and pore volume of both HZSM-5 and H[Nb]ZSM-5 are listed in Table 2. After Nb modification, the specific surface area of the zeolite decreased from 355.21 m^2^/g to 296.55 m^2^/g, and the total pore volume (V_Total_) decreased from 0.20 cm^3^/g to 0.16 cm^3^/g. This may be due to the existence of solid metal oxide aggregates on the outer surface or aggregates deposited into the pores of HZSM-5 [35,36]. This result is consistent with the literature, where others reported that incorporation of metal elements (Zn, Fe, Ca, Ce, La, Zr) into zeolites leads to a slight decrease in S_BET_ and V_Total_ [18,37].

#### 2.2.3. Chemisorption Analysis

**Figure 3:**
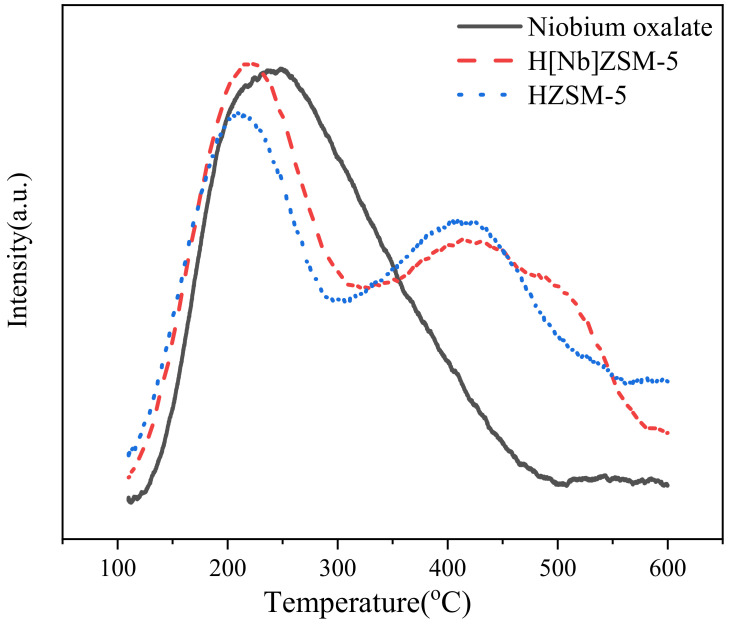
NH_3_-TPD spectra of the catalysts.

The acidic center and acidic strength have important effects on the catalytic activity of zeolite. The acidity was quantified by ammonia temperature-programmed desorption (Figure 3 and Table 3). Two peaks were observed for both HZSM-5 and H[Nb]ZSM-5 at approximately 210 °C and 410 °C, which were attributed to the weakly acidic site of non-skeletal Lewis acid and strongly acidic site of Brønsted acid, respectively. HZSM-5 displays both strong and weak acid sites, with a total amount of 0.82 mmol/g. Niobium oxalate has a weak acid site of 0.69 mmol/g. As desired, H[Nb]ZSM-5 exhibits both strong and weak acid sites and their concentration is considerably higher (0.93 mmol/g) than both HZSM-5 and niobium oxalate, especially with a relative higher content of weak acid. Obviously, doping of Nb (v) into zeolite weakened its strong Al (iii) acid sites but increased the weak Lewis acid sites. That is to say, doping niobium sites into zeolites can effectively regulate the nature and distribution of framework acidity, which would be beneficial for reducing catalyst coking and improving catalyst selectivity.

#### 2.2.4. XPS Analysis

**Figure 4:**
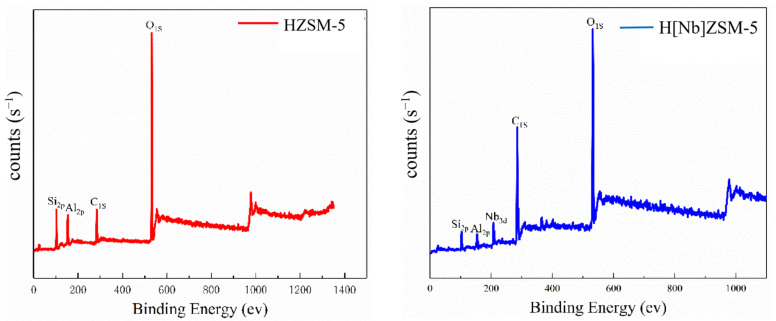
XPS full spectra of the catalysts.

The element composition of HZSM-5 and H[Nb]ZSM-5 catalyst surface was analyzed by XPS (Figure 4). HZSM-5 catalyst has three elements: Si_2p_, O_1s_, and Al_2p_. However, H[Nb]ZSM-5 had two additional peaks that appeared at 204.2–206.3 eV and 207.4–208.8 eV, which were attributed to Nb 3d_5/2_ and 3d_3/2_ core levels, respectively [34]. This indicated the successful incorporation of Nb (v) centers into the framework of catalyst.

#### 2.2.5. Scanning Electron Microscopy (SEM)/Energy-Derisive X-ray (EDX) Analysis

**Figure 5:**
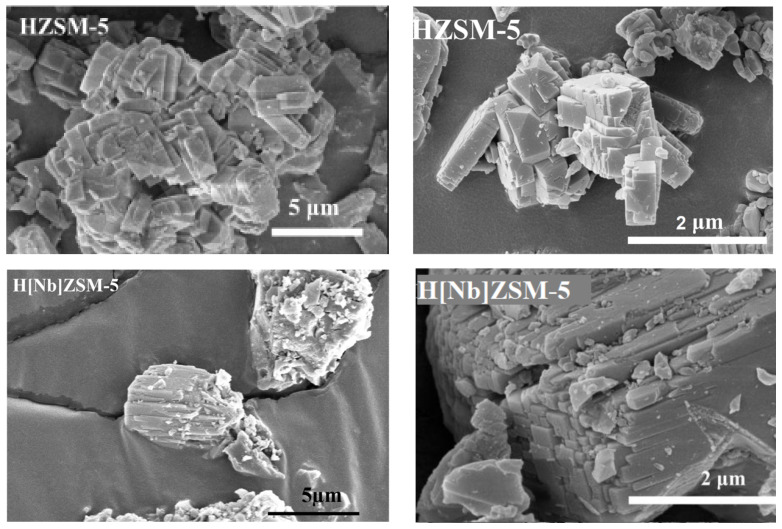
SEM images of catalysts.

HZSM-5 samples show typical cubic structures, 2.0−3.0 μm in length, 1.0−1.5 μm in width, and less than 1.0 μm in height (Figure 5). The H[Nb]ZSM-5 images reveal similar cubic structure and morphology as HZSM-5 and no obvious agglomeration of Nb particles on the surface of the cubic crystals, indicating that Nb species were highly dispersed on zeolite crystals. These are consistent with the XRD experiments. The atomic ratio of Nb/Al/Si was determined by energy-dispersive X-ray (EDX) analysis to be 0.033/0.092/1 with a homogeneous distribution of metal ions (Table 4), further confirming the successful incorporation of Nb (v) into framework.

### 2.3. Rapid Pyrolysis of Alkali Lignin

Py-GC/MS was employed for cracking and analyzing the organic compounds from alkali lignin CFP. A semi-quantitative approach was applied to determine the relative content of the detected organic compounds by analyzing the chromatographic area percentage [38].

**Figure 6:**
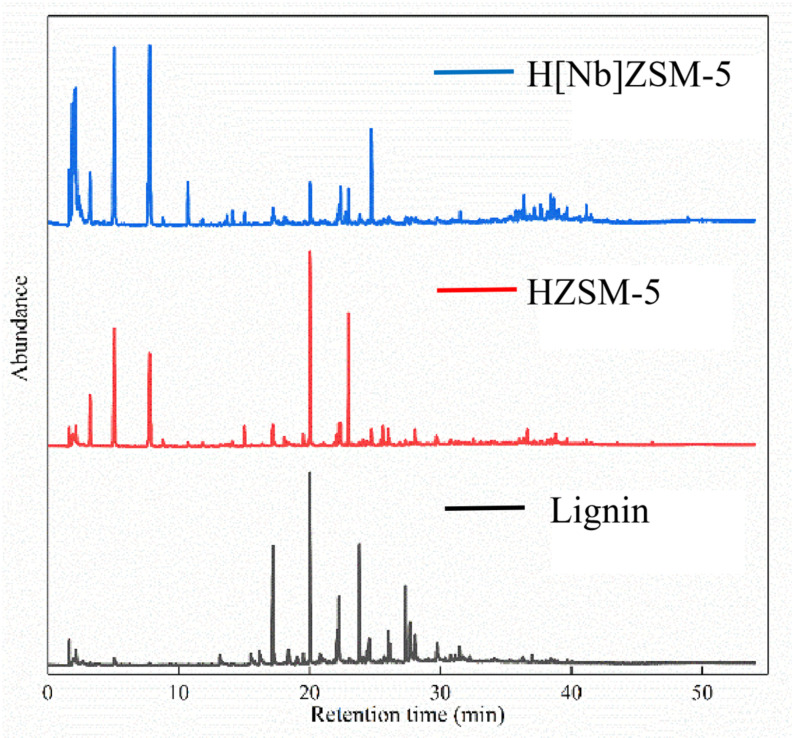
Total ion flow diagram of lignin pyrolysis products under different catalytic conditions.

**Figure 7:**
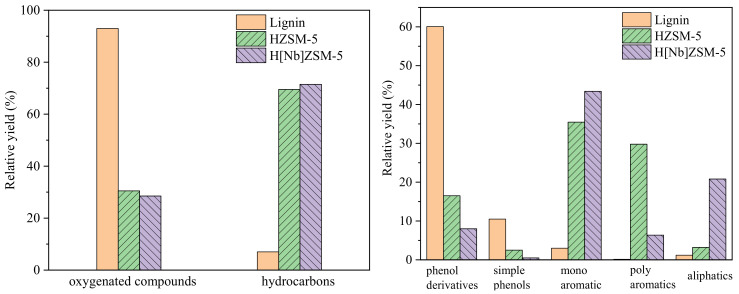
Product compositions of lignin pyrolyzed at 650 °C for 60 s with a catalyst to alkali lignin ratio of 20:1 over HZSM-5 and H[Nb]ZSM-5.

Figure 6 compares the total ion flow diagrams of condensable volatiles from non-catalytic and catalytic fast pyrolysis (CFP) operated at 650 °C for 20 s. Clearly, the presence of catalysts significantly changed the distributions and compositions of lignin pyrolysis products. Figure 7 depicts the distributions of condensable gas products. For simplicity, these pyrolysis products were classified into five groups: light phenols (with one phenolic hydroxyl group), heavy phenols (with at least two oxygen atoms), mono-aromatic hydrocarbons (MAHs, BTX), polyaromatic hydrocarbons (PAHs), and aliphatic hydrocarbons. Table 5 lists the specific compositions and contents of the aromatic hydrocarbons. Direct pyrolysis of lignin produced a large amount of oxygenates and only trace amounts of aromatic hydrocarbons. These oxygenates consist of light phenols and heavy phenols such as alkoxyphenols and alkoxyphenolic ketones. In contrast, the product distribution sharply changed when lignin pyrolysis vapors were passed over HZSM-5 or H[Nb]ZSM-5 catalyst, both producing a large amount of aromatics. Meanwhile, the decrease in the content of phenols indicated that catalysts promote the further decomposition of heavy phenolic fractions. Compared with HZSM-5, H[Nb]ZSM-5 increased the contents of MAHs and aliphatic hydrocarbons, while it decreased the contents of PAHs and phenolic compounds (simple phenols and complex phenols) in pyrolysis products. Catalyzed by H[Nb]ZSM-5, the content of MAHs and aliphatic hydrocarbons reached 43.4% and 20.8%, respectively; while under the catalysis of HZSM-5, these values were 35.5% and 3.2%, respectively. However, the contents of PAHs and phenolic compounds over HZSM-5 were approximately 29.8% and 24.9%, respectively, which were higher than the values obtained over H[Nb]ZSM-5 (6.4% PAHs and 3.2% phenols). The results indicated that the incorporation of Nb into HZSM-5 significantly improved the deoxidation performance of HZSM-5 zeolite and the generation of monoaromatic hydrocarbons.

As a bimetal catalyst possessed of both Nb (V) and Al (III) metal sites, H[Nb]ZSM-5 may promote formation of MAHs in the catalytic pyrolysis of alkali lignin by two pathways as follows. First, H[Nb]ZSM-5 retained a similar chemical composition and crystal structure, which endowed it with a shape-selective catalytic capacity as HZSM-5. In this case, the aromatics could be formed from the polymerization of olefins that were produced by either deoxygenation of oxygenates or direct fragmentation of aliphatic linkers of lignin’s aromatic units [39]. Also, it can be produced by re-polymerization of direct deoxygenation products from the primary oxygenated fragments and the intermediates. Second, the introduction of Nb (V), which has a unique electronic and energy band structure, provided H[Nb]ZSM-5 with the abilities to generate aromatics via direct phenol deoxygenation, similar to the formation of aromatics observed over CoO/MoO_3_ [17]. This also agrees with literature that reports niobium oxide-based catalysts are effective in catalytic cleavage of C–O bonds [31,32]. In conclusion, the synergistic catalytic effect of Nb (V) and HZSM-5 could cleave both sp^2^ and sp^3^ C–O–C linkages of lignin vapors and further transform the resultant oxygenates into aromatics via deoxygenation, thus improving the selectivity of monocyclic aromatic products.

Furthermore, the aromatic product contents obtained in our work were compared with those elsewhere in the literature where lignin was pyrolyzed through a similar or identical experimental device under similar conditions [31,32] (Table 6). The total aromatic hydrocarbon yield obtained from H-ZSM-5 in this study was approximately 35.5%, which is consistent with values reported in the literature (32% in ref. [13], 35.9% in ref. [40] 34.2% in ref. [26]). Hcowever, a relatively high value, 43.4%, was obtained over H[Nb]ZSM-5. It was observed that the achieved aromatic hydrocarbon yield over H[Nb]ZSM-5 is comparable with the most active reference catalysts such as HZSM-5, H-USY, niobium oxide, and niobium phosphate catalysts.

### 2.4. Catalyst Stability Evaluation

**Figure 8:**
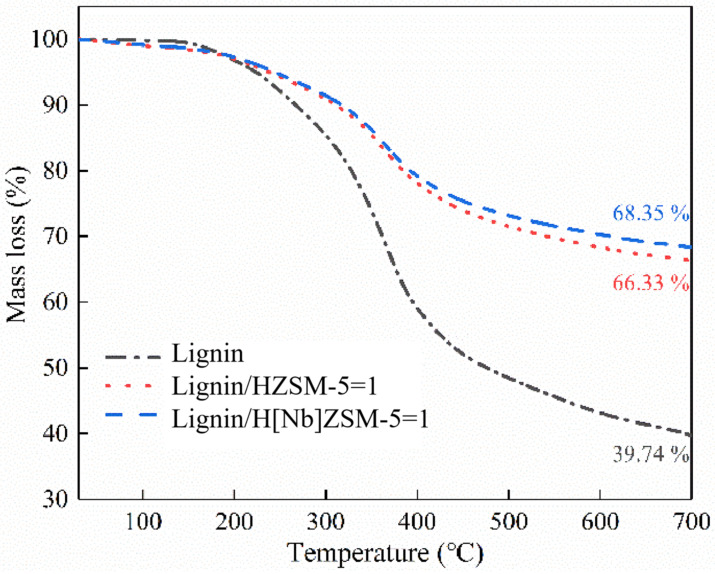
TG curves of lignin, catalyst and their mixtures (m_lignin_:m_catalyst_ = 1:1).

**Figure 9:**
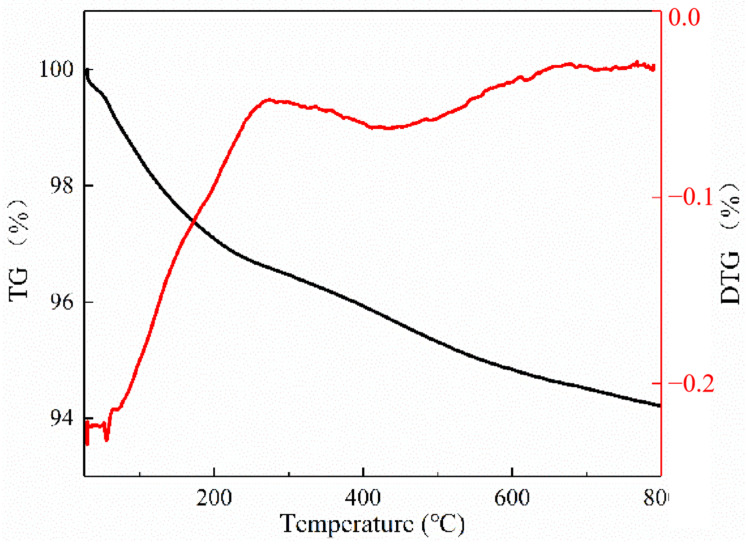
TG/DTG curves of the used H[Nb]ZSM-5 obtained in an oxygen atmosphere.

Considering the importance of catalyst stability for industrial applications, it is important to study the charring behavior of catalysts to evaluate their stability and availability in the CFP of lignin. Thus, in this study, the influence of catalysts on the formation of residual carbon during alkali lignin pyrolysis was investigated by thermo gravimetric analysis (TGA). The relevant TG curves of lignin, catalyst and their mixtures (m_lignin_:m_catalyst_ = 1:1) were depicted in Figure 8. The residual carbon of neat alkali lignin was ~39.74%. The theoretical value of residual carbon of alkali lignin in catalytic pyrolysis is 69.9%, which was calculated through simple calculations, assuming that the catalyst is stable and has no mass loss in the pyrolysis process. While actual values were 66.3% (HZSM-5) and 68.4% (H[Nb]ZSM-5). This result implies that the addition of catalyst reduced the residual carbon amount and promoted the conversion of alkali lignin to volatile fraction during the pyrolysis of alkali lignin. Then, a TG experiment of the used catalyst was carried out under an oxygen atmosphere to estimate the amount and nature of the coke deposits formed in/on catalyst (Figure 9). Two obvious weight loss stages were observed for the H[Nb]ZSM-5 catalyst. The first weight-loss interval (below 300 °C) was attributed to the loss of physically adsorbed water and low-boiling point substances, while the second weight loss stage (300 to 600 °C) resulted from the oxidative degradation of fibrous-like and graphite-like coke [41]. The fibrous-like coke can be easily removed at temperatures below 450 °C due to its filamentous and rod-like structure. But, the graphite-like coke exhibited a denser structure that required a higher oxidative degradation temperature (>450 °C) [41]. As shown in Figure 9, the TG-DTG curves indicated the dominant existence of graphite-like coke of the used H[Nb]ZSM-5 catalyst.

Further, the total, external, and internal coke quantities of the used H[Nb]ZSM-5 catalyst were calculated according to Equations (1) and (2) (Section 3.5) and are listed in Table 7. The results showed that the coke formed in lignin pyrolysis was mainly deposited on the external surface of the molecular sieve, while the quantity of internal coke formed by aromatization reaction was negligible [42]. The coke behaviors of the catalyst were related to its acid strength and acid content.

**Figure 10:**
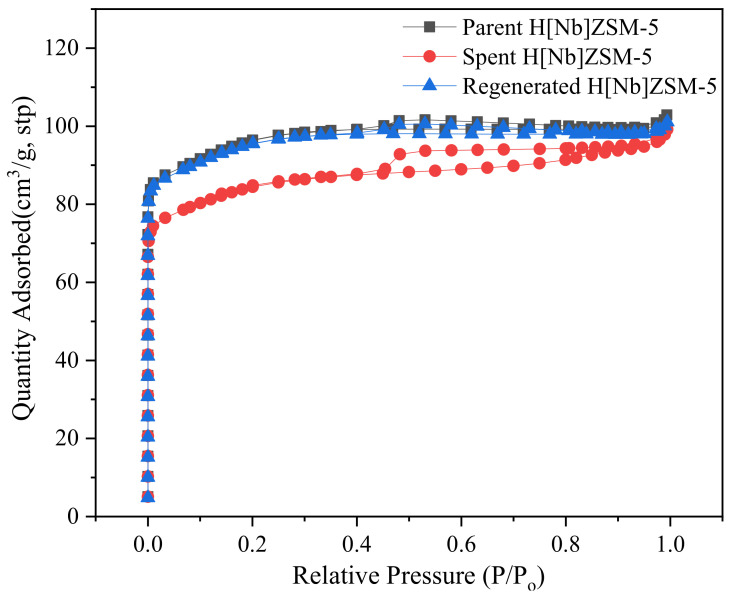
N_2_ adsorption-desorption isotherms of fresh, spent, and regenerated H[Nb]ZSM-5.

**Figure 11:**
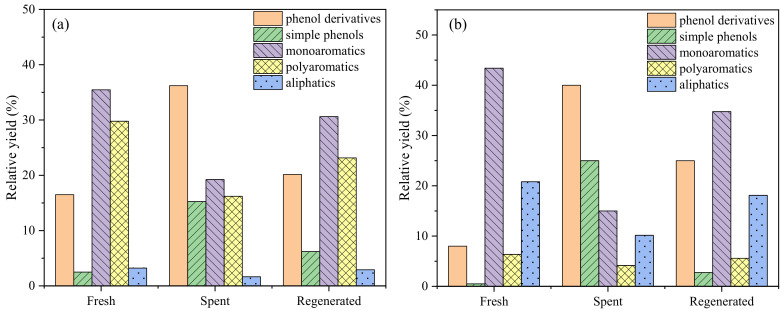
Product distributions of lignin CFP at 650 °C for 60 s over the fresh, spent, and regenerated catalysts ((**a**): HZSM-5, (**b**): H[Nb]ZSM-5).

Nitrogen adsorption experiments have been used to characterize texture characteristics of the fresh, spent, and regenerated H[Nb]ZSM-5 obtained by simply calcining of the spent catalyst under oxygen atmosphere at 500 °C (Figure 10 and Table 8). Then, three comparative tests were carried out under the conditions used for lignin pyrolysis to assess the reusability of both the HZSM-5 and H[Nb]ZSM-5 catalysts, and the product distributions were determined (Figure 11). Compared to fresh H[Nb]ZSM-5, the BET surface area, micropore surface area, and pore volume of the spent H[Nb]ZSM-5 were lower, while the average pore diameter and external surface area increased to some extent. This suggests that occurrence of surface coke deposition and pore blockage of the catalyst. Obviously, char/coke formation on/in the spent catalyst would result in the reduction of catalyst activity. In contrast to the fresh H[Nb]ZSM-5, over the spent H[Nb]ZSM-5, the contents of MAHs and PAHs decreased from 43.4% to approximately 15.0% and 6.4% to 4.2%, respectively, while the content of phenols increased from approximately 8.5% to 65.1%. However, encouragingly, these changes of catalyst in texture characteristics were almost restored after regeneration treatment. The higher content (approximately 21.2%) of aromatics and the lower content of phenols (approximately 37.3%) over regenerated H[Nb]ZSM-5 implied that the activity of the spent H[Nb]ZSM-5 had improved after regeneration. However, compared with the fresh H[Nb]ZSM-5, the activity of the spent H[Nb]ZSM-5 reduced to some extent. Similar phenomena were observed over HZSM-5 catalyst.

## 3. Materials and Methods

### 3.1. Experimental Materials

The following reagents were purchased from commercial suppliers: aluminum isopropoxide (Fuchen Co., Ltd., Tianjin, China, 98.5%), sodium hydroxide (Pengkun Co., Ltd., Tianjin, China, 96.0%), tetraethyl orthosilicate (Fuchen Co., Ltd., Tianjin, China, 98.5%), tetrapropylammonium hydroxide (Siyoupu Co., Ltd., Hefei, China, 1 mol/L), niobium oxalate (Haoxuan Co., Ltd., Guangzhou, China, 95.0%), ammonia chloride (Nankai Co., Ltd., Tianjin, China, 99.5%), and alkali lignin (Merck KGaA, Darmstadt, Germany, 99.5%.

### 3.2. Catalyst Synthesis

H[Nb]ZSM-5 and HZSM-5 were prepared using a hydrothermal method according to the literature [31]. In this study, however, niobium oxalate was used to replace the expensive and unstable niobium (V) ethanolate. In a typical synthesis, aluminium isopropoxide was first dissolved in deionized water, into which tetrapropylammonium hydroxide (TPAOH) solution as the structure-directing agent was added. The mixture was stirred at room temperature for 2 h, then niobium oxalate was added and the mixture was stirred for another 2 h. Next, tetraethyl orthosilicate was added dropwise and the mixture was stirred for another 2 h, which resulted in a gel with a chemical composition of 1Si: 0.09Al: 0.03Nb: 0.25TPAOH: 15H_2_O. The gel was then transferred to a Teflon-lined stainless-steel autoclave, which was sealed and heated to specific temperatures (110 °C, 130 °C, 150 °C, 160 °C, 170 °C, 180 °C) for certain times (24 h, 48 h, 72 h, 96 h). After centrifugation, the solid products were washed with deionized water, dried overnight at 80 °C and calcined at 550 °C under air flow for 6 h. The as-prepared solid (Na[Nb]ZSM-5) was ion exchanged with 1 mol/L ammonium chloride solution three times, followed by suction filtration, washing, drying, and roasting at 550 °C for 6 h to obtain hydrogen-type ZSM-5 molecular sieve. Throughout the entire process, sodium hydroxide was added dropwise at appropriate times to adjust the pH value. The as-prepared solid (Na[Nb]ZSM-5) was ion exchanged three times with a 1 mol/L ammonium chloride solution, followed by filtration, washing, drying, and finally calcining at 550 °C for 6 h to obtain a hydrogen-type ZSM-5 molecular sieve (H[Nb]ZSM-5). HZSM-5 sample was synthesized by the same procedure, but without the addition of niobium oxalate.

### 3.3. Catalyst Characterization

The crystal structures of the catalysts were examined using a D/max 6100 X-ray diffractometer (Shimadzu Corporation, Kyoto, Japan) with Cu Ka radiation (λ = 1.5406 Å) at 40 kV and 30 mA at a scanning rate of 8°/min from 5 to 50°. The relative crystallinity of the catalyst was calculated according to the American Society for Testing and Materials (ASTM) standard D5758-01 [43]. This test method provides a number that is the ratio of the intensity of portions of the XRD pattern of the sample to the intensity of the corresponding portion of the pattern of a reference zeolite. This intensity ratio, expressed as a percentage, is then labeled relative crystallinity. In this study, the relative crystallinity refers to the ratio of integral peak area of sample to that of the reference HZSM-5 in the range of 2θ = 22.5–25°, and calculated based on the three main diffraction peaks at 23.26°, 24.10°, and 24.56°. X-ray photoelectron spectrometer (XPS) analysis was performed on a Thermo Scientific K-Alpha (Thermo Fisher Scientific, Waltham, MA, USA) using Mg Ka as the photon source. The anode voltage was set at 15 kV and the anode current at 10 mA. The binding energies were calibrated by using the C1s peak at 284.8 eV as a reference. Field emission scanning electron microscopy and energy dispersive X-ray spectroscopy (FESEM-EDX) (Apreo S HiVac/EDX, Thermo Scientific Co., Ltd., Waltham, MA, USA) were used to study the crystal morphology, size, and elemental composition. The ratios of Nb/Al/Si were quantified using multiple regions over a sample with an Octane Elect Super detector. Nitrogen adsorption experiments were performed at 77.3 K using an Accelerated Surface Area and Porosimetry System (ASAP 2460, Micromeritics, Lutherville Timonium, MD, USA). Before the analysis, the samples were degassed at 250 ℃ for 12 h under vacuum. The specific surface area, micropore-specific surface area, and pore size distribution of the samples were obtained using multi-point BET, T-plot, and BJH methods, respectively. The acidity was evaluated using temperature-programmed desorption of ammonia (NH_3_-TPD) with an AutoChem-II-2920 chemisorption apparatus (Micromeritics, Lutherville Timonium, MD, USA). Typically, 0.1 g of catalyst sample was loaded in the quartz U-tube reactor and pre-treated under He flow at 500 °C for 1 h to remove all moisture. After cooling down to 40 °C, the sample was flushed with a NH_3_/He gas mixture (30 vol%) at a flow rate of 30 mL/min for 1 h to adsorb NH_3_. Then, the catalyst was purged with helium at 100 °C for 1 h to remove physisorbed NH_3_ from the catalyst surface. Next, the temperature rose to 600 °C at a rate of 10 °C/min while the TPD profile was recorded.

### 3.4. CFP of Lignin

**Figure 12:**
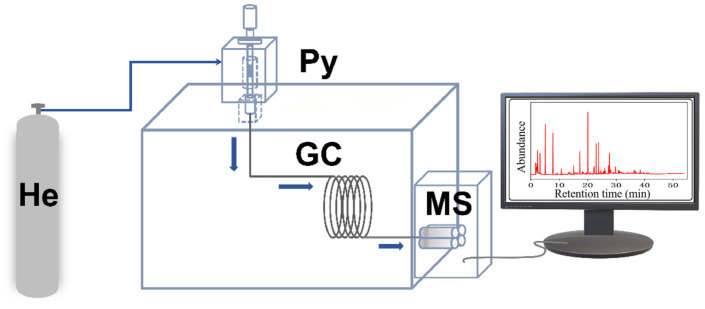
Py-GC/MS device diagram.

**Figure 13:**
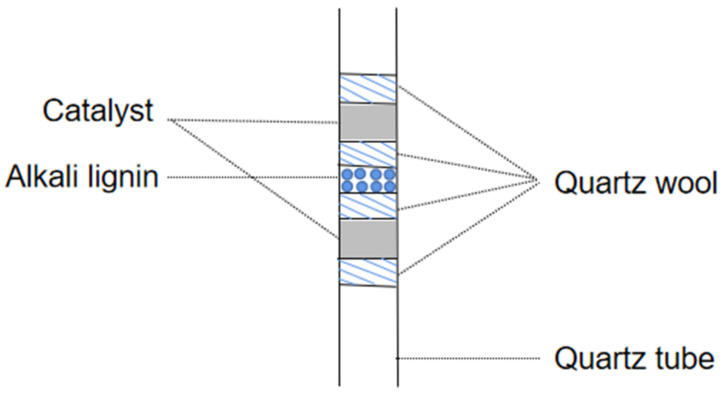
The placement of the catalyst and alkali lignin in the quartz filler tube.

Lignin, obtained from Sigma-Aldrich (USA, CAS# 8068-05-1), had the follwing elements by wt.%: C, 63.55; H, 5.49; O, 29.15; S, 1.49; N, 0.32. Catalytic fast pyrolysis of lignin was performed by coupling a pyrolyzer (CDS5200, CDS Company, Blythewood, SC, USA) to a GC–MS system (6890N/5973, Agilent Company, Santa Clara, CA, USA) (Figure 12). All pyrolyses were carried out at 650 °C, 60 s with a heating rate of 20 °C/ms. The sample was placed in the middle of the pyrolysis tube and the catalyst was next to the sample, with a sample to catalyst w:w ratio of 1:20 at both ends of the tube (Figure 13). The arrangement of sample between two catalyst layers guarantees that all vapors produced by pyrolysis of the sample pass through the catalyst bed.

The pyrolysis volatiles were transferred by high-purity helium gas into the GC/MS instrument through a transmission line, which was maintained at 285 °C. A 50:1 split ratio of the carrier gas (helium) was employed. The compressible cracking products were separated by HP-5MS quartz column (30 m × 0.25 mm × 0.25 um film thickness). The GC injector was kept at 280 °C. The GC oven was programmed to hold at 40 °C for 3 min, then heat to 270 °C for 5 min at a rate of 5 °C/min, and finally heat to 280 °C for 5 min. The mass spectrometry detector was operated in electron ionization mode (70 eV) over the m/z range from 20 to 550 amu. The ion source was kept at 230 °C. Identification of the GC-sensitive compounds was carried out by comparing with spectra of the NIST mass spectral library. Each experiment was repeated at least twice under the same condition to ensure accuracy.

A TG 209 F1 Libra thermogravimetric analyzer (NETZSCH, Selb, Germany) was used to heat 5–10 mg samples (uniformly mixed, 1:1 mass ratio of alkali lignin and catalyst) from 30 °C to 700 °C at a heating rate of 10 °C/min in argon atmosphere. TG curves were analyzed to evaluate the influence of catalyst on the amount of residual carbon from alkali lignin pyrolysis.

### 3.5. Catalyst Stability Evaluation

Thermal gravimetric analysis (TGA) was used to determine the coke behaviors of the spent catalyst. Samples weighing 10 mg were heated from 30 °C to 700 °C at a heating rate of 40 °C/min in oxygen atmosphere with a flow of 20 mL/min. The nitrogen adsorption experiment of the catalyst was also performed to investigate the effects of carbon accumulation on the catalyst-specific surface area and pore structure. The specific experimental conditions were consistent with the nitrogen adsorption experiment described in Section 3.3. For the calculation of the amount of carbon accumulation [44], it was assumed that N_2_ molecules could pass through the interconnected three-dimensional zeolite channels and fully contact the residual micropore volume of the catalyst after the reaction. The amount of internal coke was calculated based on the reduction of the micropore volume (measured by BET analysis method, cm^3^/g). The formula is as follows:

Q_MIC_ = [V_M,F_ − V_M,R_ (1 + Q_TC_)] × d_C_. (1)

The external coke quantity (i.e., the amount of coke deposited on the external surface of the catalyst) was obtained by subtracting the internal coke quantity from the total coke quantity (determined by thermogravimetric analysis curve, mg_coke_/mg_cat_). The formula is as follows:

Q_EC_ = Q_TC_ − Q_MIC_. (2)

In the two formulas above, Q_MIC_ represents the internal coke quantity; Q_EC_ is the external coke quantity; Q_TC_ is the total coke quantity; V_M,F_, V_M,R_ are the micropore volumes before and after the catalyst reaction, which were obtained from the nitrogen adsorption experiment; and d_C_ refers to the coke density, which was 1.22 g/cm^3^ (based on the C/H ratio of 1.25).

## 4. Conclusions

A niobium-doped HZSM-5 catalyst (H[Nb]ZSM-5) was constructed by hydrothermal synthesis and applied to catalytic pyrolysis of alkali lignin for preparation of monoaromatic hydrocarbons. The H[Nb]ZSM-5 catalyst fully remained within the ZSM’s crystal framework and pore structure, and the incorporation of niobium (V) and aluminum (III) endowed it with both Lewis acid and Bronsted acid sites. CFP testing showed that H[Nb]ZSM-5 catalyst promoted the generation of monoaromatic hydrocarbons and reduced the phenol content in alkali lignin pyrolysis products. H[Nb]ZSM-5 partially retained the shape selectivity of HZSM-5, while the addition of Nb active sites promoted the direct deoxygenation of phenolic products derived from alkali lignin, and inhibited the formation of polyaromatic hydrocarbons and coke. The synergistic catalytic effect of Nb (V) and HZSM-5 could cleave both sp^2^ and sp^3^ C–O–C linkages of lignin vapors and further transform the resultant oxygenates into aromatics via deoxygenation. That is to say, doping niobium sites into zeolites can effectively regulate the nature and distribution of framework acidity, which would be beneficial for reducing catalyst coking and improving catalyst selectivity. Char/coke formation on/in the spent catalyst resulted in the reduction of catalyst activity. The coke formed in lignin pyrolysis was mainly graphite-like coke, which mainly deposited on the external surface of the catalyst, while the quantity of internal coke formed by aromatization reaction was negligible. After regeneration, catalyst activity can be recovered to a large extent.

## Figures and Tables

**Table 1:** Relative crystallinity of catalyst.

Catalyst	Crystallinity (%)
HZSM-5	100.00
H[Nb]ZSM-5	87.13

**Table 2:** Texture characteristics of the catalysts.

Samples	Surface Area (m^2^/g)			Average Pore Diameter (nm)	Pore Volume (cm^3^/g)	
	BET	Micropore	External		Total	Micropore
HZSM-5	355.21	272.31	82.90	2.25	0.20	0.14
H[Nb]ZSM-5	296.55	197.36	99.19	2.15	0.16	0.11

**Table 3:** Acid contents of catalysts.

Catalyst	Acid Amount (mmol/g)		
	Weak Acid	Strong Acid	Total Acid
HZSM-5	0.26	0.56	0.82
H[Nb]ZSM-5	0.37	0.55	0.93
Niobium oxalate	0.69	/	0.69

**Table 4:** EDX result of Al (%), Si (%), Nb (%) in the catalysts.

Catalyst	Elements			Si/Al
	Al (%)	Si (%)	Nb (%)	
HZSM-5	2.06	22.49	/	10.92
H[Nb]ZSM-5	2.79	30.36	1.01	10.88

**Table 5:** The detailed compositions and contents of the aromatic products from lignin CFP at 650 °C over different catalysts for 60 s.

Aromatics	Content (Area %)		
	H[Nb]ZSM-5	HZSM-5	Non-Catalyst
Benzene	8.6	5.7	0.6
Toluene	15.3	14.7	1.1
p-Xylene	12.6	9.6	0.2
o-Xylene	3.3	2.6	0.4
Benzene, 1-ethyl-2-methyl-	2.1	1.5	0.6
Benzene, 1,2,3-trimethyl-	1.5	1.3	0.1
Indane	0.6	0.4	/
Indene	0.6	1.3	/
2-Methylindene	0.6	1.0	/
Naphthalene	2.3	13.8	/
1H-Indene, 1,3-dimethyl-	0.3	0.5	/
Naphthalene, 2-methyl-	1.2	6.4	/
Naphthalene, 1-methyl-	0.1	0.1	/
Naphthalene, 1-ethyl-	0.2	0.4	/
Naphthalene, 2,6-dimethyl-	0.2	1.0	/
Naphthalene, 1,6-dimethyl-	0.3	0.7	/
Biphenyl	/	0.3	/
1,1′-Biphenyl, 3-methyl-	/	0.2	0.1
Fluorene	/	0.7	/
1,1′-Biphenyl, 4-methyl-	/	0.3	/
9H-Fluorene, 2-methyl-	/	0.3	/
9H-Fluorene, 1-methyl-	/	0.2	/
Anthracene	0.4	1.1	/
Anthracene, 2-methyl-	/	0.8	/
Phenanthrene, 2-methyl-	/	0.4	/

**Table 6:** Fast pyrolysis of lignin into aromatics over different catalysts.

Catalyst	Feed	Reaction Conditions	Catalyst-to-Feed Ratio	Aromatic Hydrocarbon Yields (%)	References
HZSM-5	Organosolv lignin	600 °C, 1000 °C/s, 240 s	19:1	~35.9	Ref. [40]
HZSM-5	Alkaline lignin	650 °C, 20,000 °C/s, 20 s	4:1	~32.0	Ref. [13]
H-USY	Alkaline lignin	650 °C, 20,000 °C/s, 20 s	4:1	~40	Ref. [13]
HZSM-5	Kraft lignin	650 °C, 20,000 °C/s, 20 s	4:1	~34.2	Ref. [26]
Nb_2_O_5_	Kraft lignin	650 °C, 20,000 °C/s, 20 s	4:1	~28.1	Ref. [26]
Niobium phosphate	Kraft lignin	650 °C, 20,000 °C/s, 20 s	4:1	~35.1	Ref. [26]
H[Nb]ZSM-5	Alkaline lignin	650 °C, 20,000 °C/s, 20 s	20:1	~43.4	This study

**Table 7:** Carbon accumulation analysis of the spent H[Nb]ZSM-5 catalyst.

Catalyst	Coke Formation (mg_coke_/g_cat_)		
	Q_TC_	Q_MIC_	Q_EC_
Spent H[Nb]ZSM-5	56.49	5.62	50.87

**Table 8:** Texture characteristics of fresh, spent, and regenerated H[Nb]ZSM-5.

Samples	Surface Area (m^2^/g)			Average Pore Diameter (nm)	Pore Volume (cm^3^/g)	
	BET	Micropore	External		Total	Micropore
Fresh H[Nb]ZSM-5	296.55	197.36	99.19	2.15	0.159	0.101
Spent H[Nb]ZSM-5	286.48	178.03	108.45	2.37	0.153	0.096
Regenerated H[Nb]ZSM-5	292.95	196.56	96.38	2.14	0.156	0.105

## Data Availability

The data presented in this study are available on request from the corresponding author.

## Supplementary Materials

The following supporting information can be downloaded at: https://www.mdpi.com/article/10.3390/molecules28104245/s1, Figure S1: XRD patterns and relative crystallinity of H[Nb]ZSM-5 synthesized at different temperatures; Figure S2: XRD patterns and relative crystallinity of H[Nb]ZSM-5 synthesized at different time; Figure S3: XRD patterns and relative crystallinity of H[Nb]ZSM-5 synthesized at different alkalinity; Figure S4: XRD patterns of H[Nb]ZSM-5 synthesized with different niobium sources.

## Sample Availability

Samples of the compounds are available from the authors.
