# Supplementary material for: Efficient Conversion of Lignin to Aromatics via Catalytic Fast Pyrolysis over Niobium-Doped HZSM-5
Source: Molecules. 2023 May 22;28(10):4245. doi: 10.3390/molecules28104245 (PMC10222616; doi:10.3390/molecules28104245)
Supplement: Supplementary file 1 [file molecules-28-04245-s001.zip › molecules-2364086-supplementary.pdf]

1. Synthesis of H[Nb]ZSM-5 catalyst

1.1 Influence of crystallization temperature

The effect of crystallization temperature on the crystallization process of the bimetallic catalyst H[Nb]ZSM-5 was investigated at crystallization time of 72 h and PH=12. Figure S1 showed the XRD spectra and relative crystallinity of the synthesized samples at different temperatures. Products obtained at 110 °C were almost amorphous. When the temperature above 130 °C, the HZSM-5 characteristic peak appeared and the relative crystallinity increased with the increase of temperature. The product's relative crystallinity obtained at 150 °C and 160 °C, respectively reached to about 79.1% and 88.16%. But, the relative crystallinity of the product decreased to 68.36% at 180 °C. It showed that crystallization temperature was a key factor to control the relative crystallinity of H[Nb]ZSM-5. So, 170 °C was suitable temperature for the synthesis of H[Nb]ZSM-5 with a high relative crystallinity.

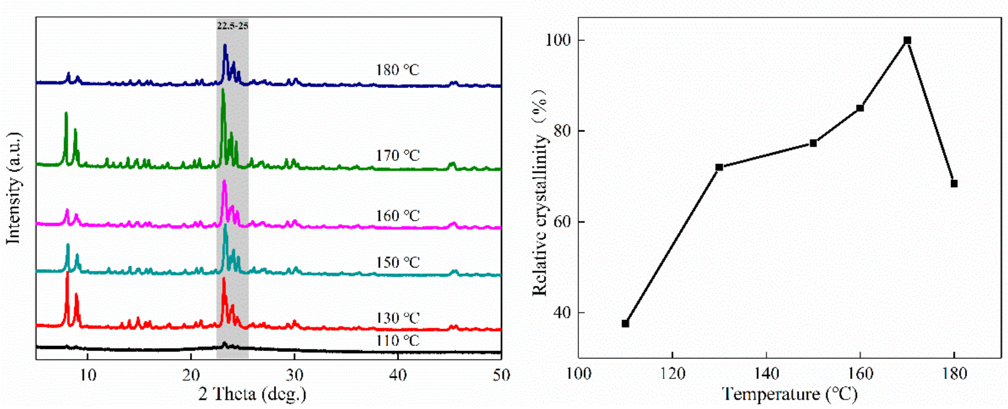

Figure S1. XRD patterns and relative crystallinity of H[Nb]ZSM-5 synthesized at different temperatures.

1.2 Influence of crystallization time

The effect of crystallization time on the crystallization process of H[Nb]ZSM-5 was investigated at 170°C and PH=12. Figure S2 showed the XRD spectra and relative crystallinity of the synthesized samples at different time.

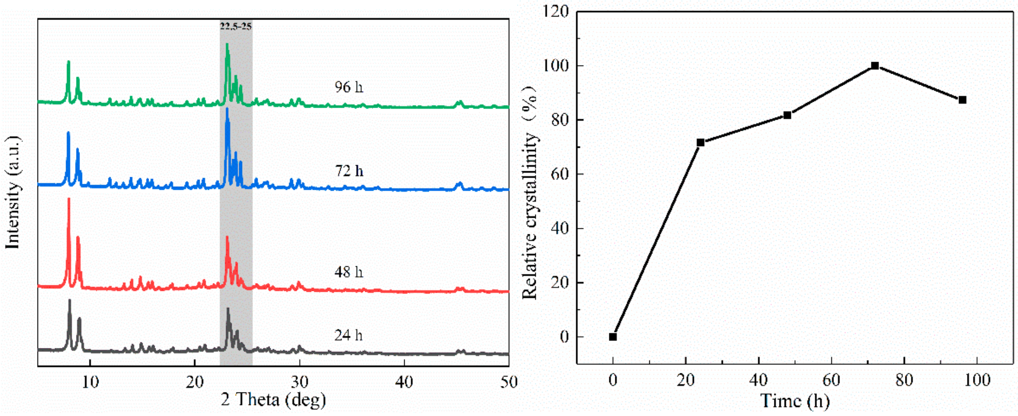

Figure S2. XRD patterns and relative crystallinity of H[Nb]ZSM-5 synthesized at different time.

It can be seen that the relative crystallinity of the products increased with the increase of crystallization time from 24 h to 72 h, and reached a maximum of 100% at 72 h. When the crystallization time was further extended to 96 h, the relative crystallinity of the product decreased to 87.27%. When the crystallization time is too long, the concentration of Si and Al in liquid phase and gel phases decreased greatly. This may make Si and Al in the molecular sieve re-dissolve into the solution, leading a decrease of the crystallinity of molecular sieve. Therefore, 72 h was selected as the optimum crystallization time for H[Nb]ZSM-5.

1.3 Influence of alkalinity

The effect of alkalinity on the crystallization of H[Nb]ZSM-5 was investigated at 170°C and 72 h under different alkalinity. Figure S3 shows the XRD spectra and relative crystallinity of the synthesized samples.

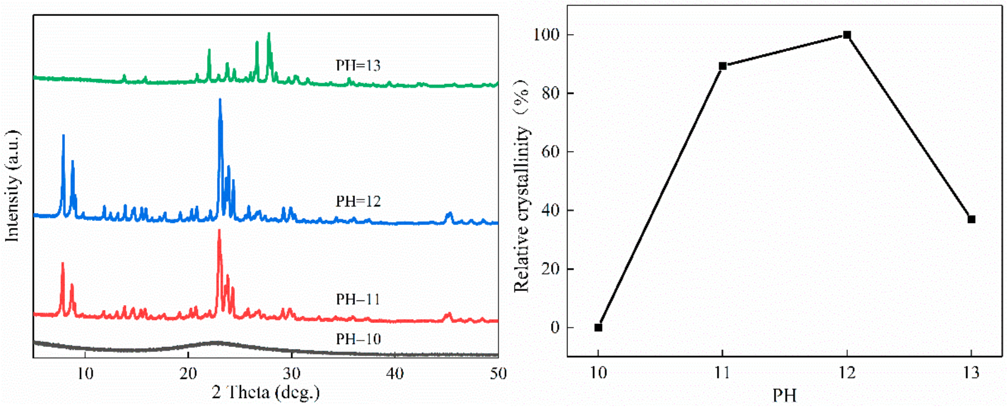

Figure S3. XRD patterns and relative crystallinity of H[Nb]ZSM-5 synthesized at different alkalinity.

As can be seen from Figure S3, when the alkalinity was low (pH=10), the secondary structure of HZSM-5 zeolite could not be polycondensated by silicon and aluminum sources, resulting in an amorphous product. When pH=10-12, the relative crystallinity of the product increased with the increase of pH, and reached the maximum at pH=12. But, the relative crystallinity of the product decreased to 37.00% when pH=13. This is because that high alkalinity could lead to crystalline transformation. In turn, it reduced the relative crystallinity of the product. In conclusion, the optimum pH for the synthesis of H[Nb]ZSM-5 was 12.

1.3 Influence of niobium source

The effect of niobium source on the crystallization process of H[Nb]ZSM-5 was investigated at 170°C and PH=12. The XRD spectra of the samples synthesized using niobium oxalate and ethanol niobium, were showed in Figure S4, separately. It found that under the same experimental conditions, the crystallinity of the target product synthesized from niobium ethanol as a niobium source is lower than that of niobium oxalate as a niobium source, with a relative crystallinity of only 41.18%. This may be attributed to the high susceptibility of niobium ethanol to deliquescence and its low stability during the reaction. Therefore, the use of niobium oxalate as the niobium source for the synthesis of H[Nb]ZSM-5 is more appropriate, taking into account both economic cost and yield factors.

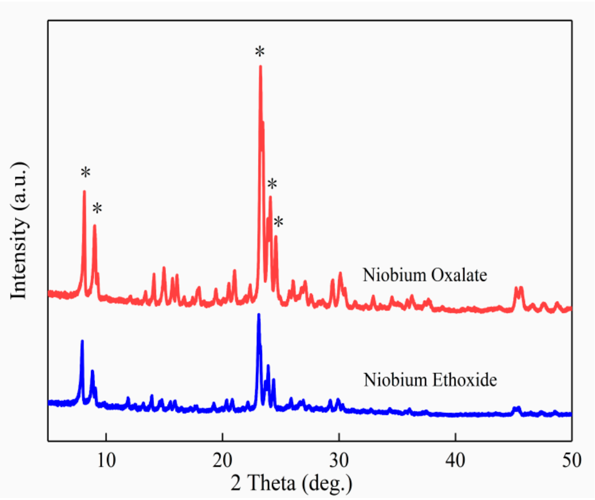

Figure S4. XRD patterns of H[Nb]ZSM-5 synthesized with different niobium sources. (*Peaks used for calculating relative crystallinity).
